# VAS2870 and VAS3947 attenuate platelet activation and thrombus formation via a NOX-independent pathway downstream of PKC

**DOI:** 10.1038/s41598-019-55189-5

**Published:** 2019-12-11

**Authors:** Wan Jung Lu, Jiun Yi Li, Ray Jade Chen, Li Ting Huang, Tzu Yin Lee, Kuan Hung Lin

**Affiliations:** 1 Department of Medical Research, Taipei Medical University Hospital, 110 Taipei, Taiwan; 2 Department of Pharmacology, School of Medicine, College of Medicine, Taipei Medical University, 110 Taipei, Taiwan; 3 Graduate Institute of Metabolism and Obesity Sciences, College of Nutrition, Taipei Medical University, 110 Taipei, Taiwan; 4 Department of Surgery, Mackay Memorial Hospital, 104 Taipei, Taiwan; 5 Department of Medicine, Mackay Medical College, 252 New Taipei City, Taiwan; 6 Division of General Surgery, Department of Surgery, Taipei Medical University Hospital, 110 Taipei, Taiwan; 7 Department of Surgery, School of Medicine, College of Medicine, Taipei Medical University, 110 Taipei, Taiwan; 8 Graduate Institute of Medical Sciences, College of Medicine, Taipei Medical University, 110 Taipei, Taiwan; 9 Institute of Biomedical Sciences, Mackay Medical College, 252 New Taipei City, Taiwan

**Keywords:** Platelets, Platelets

## Abstract

NADPH oxidase (NOX) enzymes are involved in a various physiological and pathological processes such as platelet activation and inflammation. Interestingly, we found that the pan-NOX inhibitors VAS compounds (VAS2870 and its analog VAS3947) exerted a highly potent antiplatelet effect. Unlike VAS compounds, concurrent inhibition of NOX1, 2, and 4 by treatment with ML171, GSK2795039, and GKT136901/GKT137831 did not affect thrombin and U46619-induced platelet aggregation. These findings suggest that VAS compounds may inhibit platelet aggregation via a NOX-independent manner. Thus, we aimed to investigate the detailed antiplatelet mechanisms of VAS compounds. The data revealed that VAS compounds blocked various agonist-induced platelet aggregation, possibly via blocking PKC downstream signaling, including IKKβ and p38 MAPK, eventually reducing platelet granule release, calcium mobilization, and GPIIbIIIa activation. In addition, VAS compounds inhibited mouse platelet aggregation-induced by collagen and thrombin. The *in vivo* study also showed that VAS compounds delayed thrombus formation without affecting normal hemostasis. This study is the first to demonstrate that, in addition to inhibiting NOX activity, VAS compounds reduced platelet activation and thrombus formation through a NOX-independent pathway downstream of PKC. These findings also indicate that VAS compounds may be safe and potentially therapeutic agents for treating patients with cardiovascular diseases.

## Introduction

Platelets play a crucial role in normal hemostasis, and also involve in pathological conditions such as inflammation, tumor metastasis, atherosclerosis, and stroke^1,2^. When blood vessels are injured, circulating platelets adhere to the injured site of the vessel, and are activated by the exposed extracellular matrix, such as collagen and von Willebrand factor (vWF), that can be partly mediated through the interaction of collagen-glycoprotein VI (GPVI) and vWF-GPIb-V-IX. Activated platelets can further recruit more circulating platelets and initiate a coagulation cascade to produce thrombin and fibrin. These processes can lead to a firm platelet plug formation at the injury site to achieve hemostasis^1,2^. However, uncontrolled platelet activation and aggregation under pathological conditions may result in thrombus formation and subsequent vessel occlusion. Thus, appropriate regulation of platelet function is necessary to prevent thrombus formation during these thrombosis-prone conditions.

NADPH oxidase (NOX) enzymes are the major sources of reactive oxygen species (ROS), and they are involved in various physiological and pathological processes such as immunity, inflammation, atherosclerosis, diabetic nephropathy, and cancer^3,4^. The NOX family consists of seven members: NOX1–5 and two dual oxidases (Duox), namely Duox1 and Duox2. Although NOX1, 2, 4, and 5 have been implicated in vascular diseases^5^, only NOX1 and 2, but not NOX4 and 5, are present in human platelets^6^. These findings suggest that NOX1 and 2 play more crucial roles in regulating platelet function. A study reported that NOX-derived ROS enhanced platelet aggregation and platelet-dependent thrombosis^7^. NOX-derived O_2_^−^ may promote platelet activation through phospholipase A_2_-dependent arachidonic acid formation. NOX-derived O_2_^−^ can also be rapidly converted into H_2_O_2_ by superoxide dismutase, which in turn activates platelets through calcium mobilization. Furthermore, NOX-derived ROS can inactivate nitric oxide^7^. These effects of NOX-derived ROS lead to the amplification of platelet activation. Moreover, experimental and clinical studies have reported that NOX1 and 2 are involved in platelet activation and thrombus formation^6–11^. Delaney *et al*.^9^ reported that NOX1 plays a selective role in G protein-coupled receptor (GPCR)-induced platelet aggregation and secretion, whereas NOX2 plays crucial roles in both GPCR- and GPVI-dependent platelet aggregation and secretion. By contrast, Walsh *et al*.^11^ demonstrated that gene deletion of NOX2 did not affect GPVI-dependent platelet aggregation and secretion induced by the collagen-related peptide (CRP, a specific agonist of GPVI). Furthermore, Walsh *et al*.^11^ and Vara *et al*.^6^ have reported that NOX1, but not NOX2, regulates GPVI-mediated platelet activation. Although mechanisms through which NOX1 and NOX2 regulate platelet activation remain controversial, both NOX1 and NOX2 play crucial roles in platelet activation. Therefore, targeting NOX may be a potential therapeutic strategy for treating patients with cardiovascular diseases.

To discover drugs or compounds that may be applied in clinical settings, several NOX inhibitors were used in the present study. We found that a small-molecule, specific, and nonselective inhibitor of NOX, VAS2870, or its analog, VAS3947, exert a potent antiplatelet effect. Although a previous study has reported that VAS2870 prevent thrombin- and CRP-induced platelet aggregation^12^, its detailed mechanism remains unclear. However, intriguingly, our preliminary results revealed that the pan-NOX inhibitors VAS compounds (VAS2870 and its analog VAS3947) exerted a highly potent antiplatelet effect. Unlike VAS compounds, concurrent inhibition of NOX1, 2, and 4 by treatment with ML171, GSK2795039, and GKT136901/GKT137831 did not affect thrombin and U46619-induced platelet aggregation. These findings suggest that, in addition to serving as NOX inhibitors, VAS compounds may inhibit platelet aggregation via a NOX-independent manner. Therefore, we further determined the detailed mechanisms underlying the antiplatelet effect of VAS2870 and VAS3947.

## Results

### VAS compounds inhibits platelet aggregation induced by various platelet agonists

**Figure 1:**
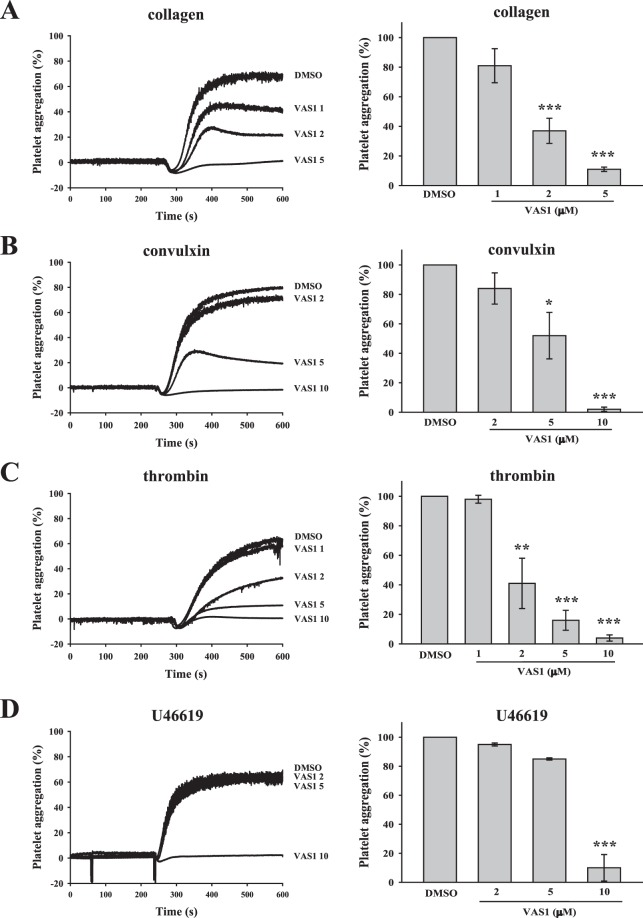
VAS1 mediated the inhibition of platelet aggregation in human platelets stimulated by various agonists. Washed platelets (3.6 × 10^8^ cells/ml) were preincubated with DMSO (solvent control) or VAS1 (VAS2870, 1–10 μM) following the stimulation with (**A**) collagen (1 μg/ml), (**B**) convulxin (10 ng/ml), (**C**) thrombin (0.02 U/ml) and (**D**) U46619 (1 μM) to trigger platelet aggregation. Left panels indicate the tracing curve of platelet aggregation, and right panels indicate the statistical bar graphs of platelet aggregation (%). Data (**A**–**D**) are presented as means ± SEM (*n* = 3). *p < 0.05, **p < 0.01, and ***p < 0.001, compared with the DMSO (solvent control) group. Comparisons were made by ANOVA.

**Figure 2:**
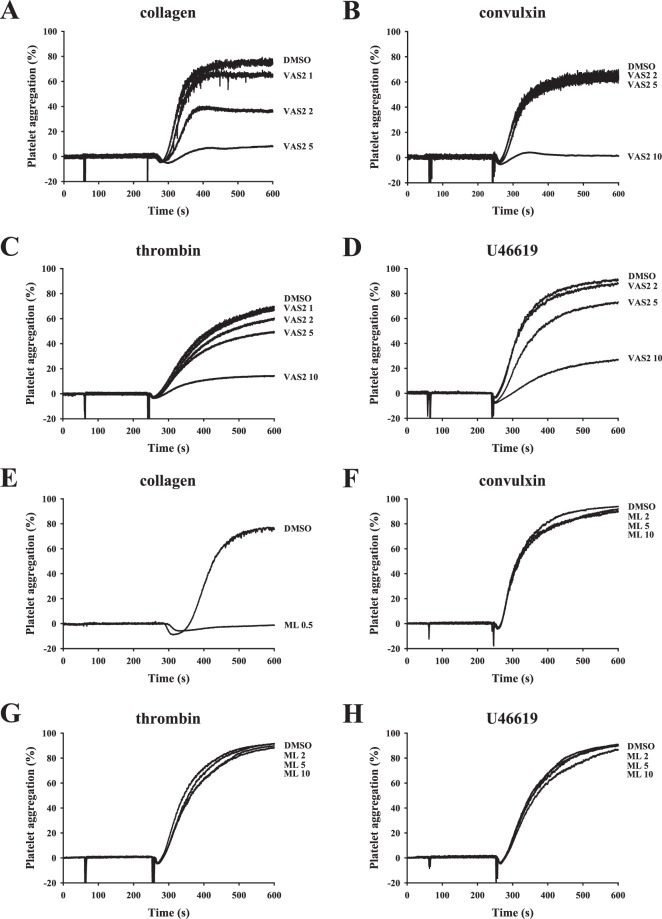
Effects of VAS2 and ML171 on platelet aggregation induced by various agonists. Washed platelets (3.6 × 10^8^ cells/ml) were pre-incubated with DMSO (solvent control), (**A**–**D**) VAS2 (VAS3947, 1–10 μM), or (**E**–**H**) ML171 (ML, 0.5–10 μM) following the stimulation of collagen (1 μg/ml), convulxin (10 ng/ml), thrombin (0.02 U/ml) and U46619 (1 μM) to trigger platelet aggregation. Profiles (**A**–**H**) are representative examples of three similar experiments.

As shown in Fig. 1A, the pan-NOX inhibitor VAS2870 (VAS1) completely inhibited collagen-induced platelet aggregation at the concentration of 5 μM. Moreover, VAS1 at a concentration of 10 μM completely abolished platelet aggregation induced by convulxin, thrombin, or U46619, indicating that VAS1 effectively inhibits platelet activation (Fig. 1B–D). Furthermore, VAS3947 (VAS2), an analog of VAS1, exert effects similar to those exerted by VAS1 on platelet aggregation (Figs. 2A–D; S1A). These results suggest that VAS compounds can inhibit GPCR- and GPVI-mediated platelet activation.

**Figure 3:**
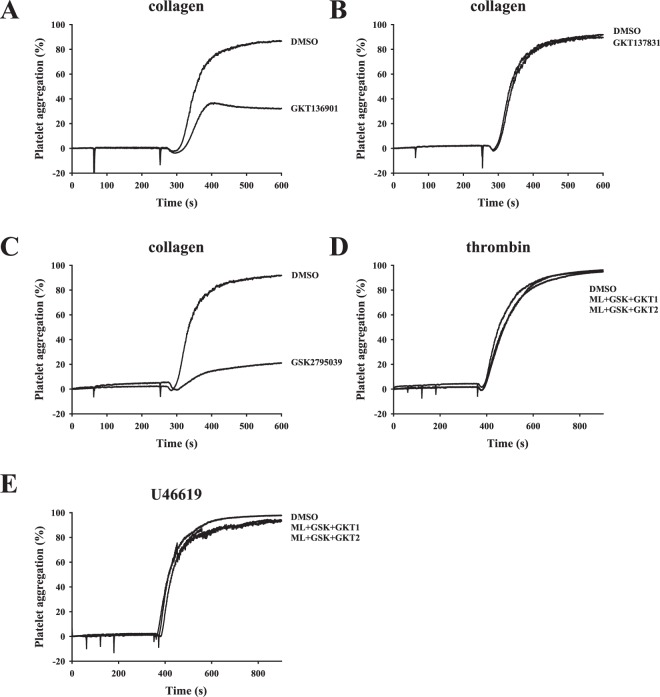
Effects of selective and nonspecific NOX inhibitors on platelet aggregation. Washed platelets (3.6 × 10^8^ cells/ml) were pre-incubated with DMSO (solvent control), (**A**) GKT136901 (GKT1, 20 μM), (**B**) GKT137831 (GKT2, 20 μM), (**C**) GSK2795039 (GSK, 20 μM), or (**D**,**E**) combination of ML171 (ML, 0.5 μM), GSK (20 μM), and GKT1 or GKT2 (20 μM) following stimulation with collagen (1 μg/ml), convulxin (10 ng/ml), thrombin (0.02 U/ml) and U46619 (1 μM) to trigger platelet aggregation. Profiles (**A**–**E**) are representative examples of three similar experiments.

In this study, we also used the selective NOX1 inhibitor ML171. Similar to the finding of a previous study^6^, we found that ML171 (0.5 μM) markedly inhibited collagen-induced platelet aggregation (Fig. 2E). However, ML171, even at high concentrations of 10 and 100 μM, did not exert an inhibitory effect on platelet aggregation induced by convulxin, thrombin, or U46619 (Figs. 2F–H; S1B–E), indicating that ML171 is sensitive to the inhibition of collagen-mediated platelet activation. We also used the NOX1/4 inhibitors GKT136901 and GKT137831. Results showed that GKT136901 (20 μM), but not GKT137831 (20 μM), could inhibit collagen-induced platelet aggregation (Fig. 3A,B), and both the compounds, even at a high concentration of 100 μM, did not affect convulxin- and thrombin-induced platelet aggregation (Fig. S2A,B). Although, GKT136901 (100 μM), but not GKT137831 (100 μM), completely blocked U46619-induced platelet aggregation (Fig. S2C,D), it is 10-fold less potent than VAS compounds. Moreover, unlike VAS compounds, GKT136901 (100 μM) also did not affect convulxin- and thrombin-induced platelet aggregation. On the other hand, our data also revealed that the selective NOX2 inhibitor GSK2795039 (20 μM) effectively inhibited collagen-induced platelet aggregation (Fig. 3C) but not convulxin-, thrombin-, or U46619-induced platelet aggregation even at a high concentration of 100 μM (Fig. S3). Moreover, concurrent inhibition of NOX1, 2, and 4 by treatment with ML171, GSK2795039, and GKT136901/GKT137831 did not affect thrombin and U46619-induced platelet aggregation (Fig. 3D,E). These findings suggest that, in addition to serving as NOX inhibitors, VAS compounds may inhibit platelet aggregation via a NOX-independent manner. In addition, the nonspecific NOX inhibitors diphenyleneiodonium (DPI) and apocynin (APO) were used. As shown in Fig. S4, DPI (100 μM) and APO (500 μM) effectively reduced collagen- and convulxin- induced platelet aggregation, but not thrombin- and U46619-induced platelet aggregation. Therefore, we further investigated the detailed mechanisms underlying the antiplatelet effect of VAS compounds in the subsequent experiments.

### VAS compounds block platelet activation through the inhibition of protein kinase C downstream signaling pathway

**Figure 4:**
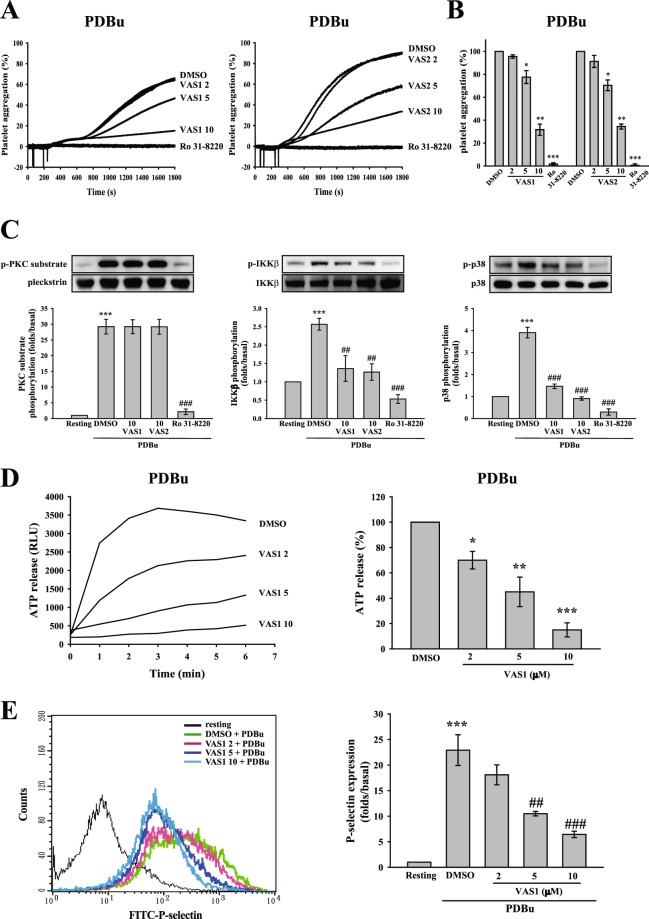
Effects of VAS compounds on the PKC/NOX downstream signaling pathway. (**A**) Washed platelets (3.6 × 10^8^ cells/ml) were pre-incubated with DMSO (solvent control), VAS compounds (2–10 μM), or Ro 31-8220 (2 μM) following stimulation with PDBu (150 nM) to trigger platelet aggregation. (**B**) The statistical analysis in (**A**). (**C**) After the reaction, platelet lysates were directly collected, and then subjected to Western blotting. Specific antibodies were used to detect PKC, IKKβ, and p38 MAPK. (**D**,**E**) Luciferase/luciferin and FITC-P-selectin antibody were used to detect ATP release and P-selectin using a microplate reader and flow cytometry, respectively. Data (**B**,**D**) are presented as means ± S.E.M. (*n = *3). *p < 0.05, **p < 0.01, and ***p < 0.001, compared with the DMSO (solvent control) group. Data (**C**,**E**) are presented as means ± SEM (**C**, *n* = 4; **E**, *n* = 3). ***p < 0.001, compared with the resting group. ^##^p < 0.01 and ^###^p < 0.001, compared with the PDBu-treated (positive control) group. Comparisons were made by ANOVA.

VAS compounds inhibited GPVI and GPCR-induced platelet aggregation, indicating that VAS compounds may inhibit a common pathway of platelet activation. The protein kinase C (PKC) pathway is a well-known common pathway involved in platelet activation. Thus, we first determined whether VAS compounds affect PKC signaling. As shown in Fig. 4A,B, VAS compounds (5–10 μM) and the PKC inhibitor Ro 31-8220 effectively reduced platelet aggregation induced by the PKC activator phorbol 12,13-dibutyrate (PDBu), suggesting that VAS compounds may inhibit PKC or its downstream signaling. In addition, ML171, GKT compounds, GSK2795039, DPI, and APO were also performed, and the data revealed that these inhibitors did not affect PDBu-induced platelet aggregation (Fig. S5). To confirm whether VAS compounds inhibit PKC activation, the phosphorylation of p47 protein (pleckstrin), a major PKC substrate (~47 kD) that is widely used to determine PKC activity, was detected through Western blotting. As shown in Fig. 4C, Ro 31-8220, but not VAS1 and VAS2, markedly prevented PDBu-induced p47 phosphorylation, indicating that VAS compounds do not directly inhibit PKC activity but may inhibit PKC downstream signaling. Thus, we also determine the phosphorylation of IKKβ and p38 MAPK, which have been reported to conduct PKC-mediated granule release and thromboxane A_2_ secretion, respectively^13–15^. The data showed that VAS compounds as well as Ro 31-8220 significantly inhibit the phosphorylation of IKKβ and p38 MAPK. These findings also suggest that VAS compounds attenuated platelet activation, at least in part, through the inhibition of PKC downstream signaling pathway, including IKKβ and p38 MAPK.

### VAS compounds attenuate PDBu-induced granule release, calcium mobilization, and glycoprotein IIbIIIa activation

**Figure 5:**
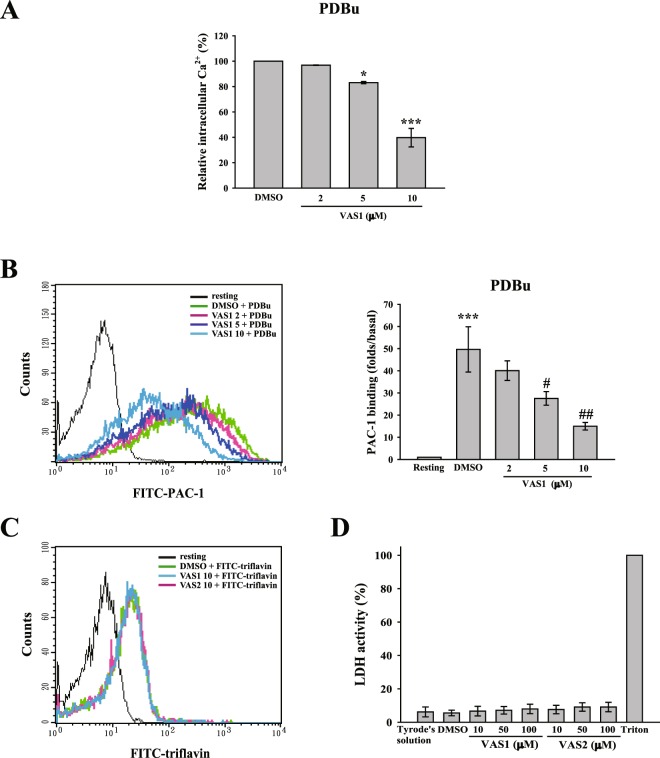
Effects of VAS compounds on calcium mobilization and GPIIbIIIa activation. (**A**,**B**) Washed platelets were pre-incubated with DMSO (solvent control) and VAS1 (2–10 μM) before the addition of PDBu to trigger platelet activation. Fura-2 and FITC-PAC1 antibodies were used to detect calcium mobilization and GPIIbIIIa activation by a microplate reader and flow cytometry, respectively. (**C**) FITC-triflavin was used to observe the binding capacity of VAS compounds (10 μM) on the GPIIbIIIa receptor through flow cytometry. (**D**) LDH assay kits were used to determine the cytotoxicity of VAS compounds (10–100 μM) on platelets. The Triton-treated group as maximum toxicity (100%). Data (**A**) are presented as means ± SEM (*n = *3). *p < 0.05 and ***p < 0.001, compared with the DMSO (solvent control) group. Data (**B**) are presented as means ± S.E.M. (*n = *3). ***p < 0.001, compared with the resting group. ^#^p < 0.05 and ^##^p < 0.01, compared with the PDBu-treated (positive control) group. Comparisons were made by ANOVA. Profiles (**C**) are representative examples of three similar experiments.

In the present study, several indicators of platelet activation induced by PDBu were determined. As shown in Fig. 4D,E, VAS1 (5–10 μM) significantly inhibited PDBu-induced ATP release and P-selectin secretion, suggesting that VAS1 reduced PKC-mediated platelet granule release. VAS1 (5–10 μM) also blocked PDBu-induced calcium mobilization and GPIIbIIIa activation (Fig. 5A,B). Likely, VAS2 also have inhibitory effects similar to those exerted by VAS1 on platelet activation (Fig. S6A–D). These findings support that VAS compounds prevent platelet activation through the PKC downstream signaling pathway.

We considered that VAS compounds may directly block GPIIbIIIa, the final step of platelet aggregation, thereby inhibiting various agonists-stimulated platelet aggregation. Therefore, we further determined whether VAS compounds interfere with GPIIbIIIa. We found that VAS1 and VAS2 did not affect the binding of triflavin, a GPIIbIIIa antagonist^16^, to GPIIbIIIa (Fig. 5C), indicating that VAS compounds do not block GPIIbIIIa. In addition, the result of lactate dehydrogenase (LDH) assay revealed that VAS compounds (10–100 μM) did not exhibit cytotoxicity in human platelets (Fig. 5D), suggesting that the potent antiplatelet activity of VAS compounds is not caused by damaging platelets.

### VAS compounds prevent platelet aggregation *in vitro* and thrombus formation *in vivo* in mice

**Figure 6:**
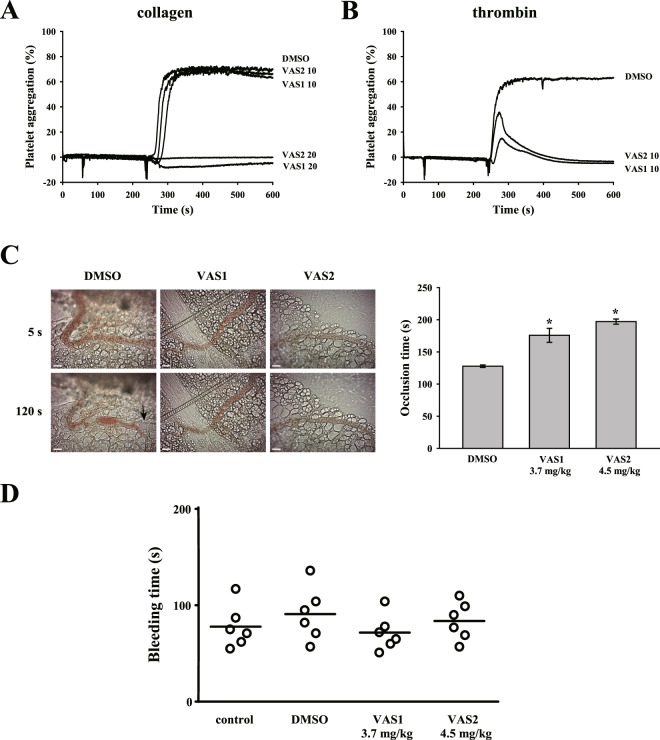
Effects of VAS compounds on platelet aggregation, thrombus formation, and hemostasis in mice. Washed mouse platelets (1 × 10^8^ cells/ml) were preincubated with DMSO (solvent control) or VAS compounds (10–20 μM) following stimulation with 1 μg/ml collagen (**A**) and 0.02 U/ml thrombin (**B**) to trigger platelet aggregation. (**C**) Mice received an intravenous bolus of DMSO, VAS1 (3.7 mg/kg) or VAS2 (4.5 mg/kg), and their mesenteric venules were irradiated to induce microthrombus formation. The arrow indicates occlusion of the mesenteric venule. The scale bar indicates 30 μm. (**D**) Bleeding was induced by severing the tail at 3 mm from the tail tip, and the bleeding tail stump was immersed in saline. Subsequently, the bleeding time was continually recorded until no sign of bleeding was observed for at least 10 s. Each point in the scatter plots graph represents a mouse (*n* = 6). Profiles (**A**,**B**) are representative examples of three similar experiments. Data (**C**) are presented as the mean ± SEM (*n = *6). *p < 0.05, compared with the DMSO group. Comparisons were made by ANOVA.

We further determined the effect of VAS compounds on platelet activation and thrombus formation in mice. We observed that VAS compounds (10–20 μM) reduced platelet aggregation induced by collagen and thrombin in mouse platelets (Fig. 6A,B).

In the *in vivo* model, fluorescein sodium was used to evaluate platelet thrombus formation in mesenteric microvessels; this model was exposed to UV irradiation, which damaged the endothelium and subsequently caused vascular occlusion. The occlusion time was recorded using a real-time monitor. As shown in Fig. 6C, the dimethyl sulfoxide (DMSO) group had an occlusion time of approximately 127.8 s. Compared with the DMSO treatment, VAS1 (3.7 mg/kg) and VAS2 (4.5 mg/kg) treatments prolonged the occlusion time by 50.0 and 69.3 s (both *p* < 0.01, *n* = 8), respectively. Moreover, VAS compounds did not affect normal hemostasis, as evidenced from the assay of tail-bleeding time (Fig. 6D). These findings indicate that VAS compounds exerted antithrombotic effects without side effect of bleeding.

## Discussion

**Figure 7:**
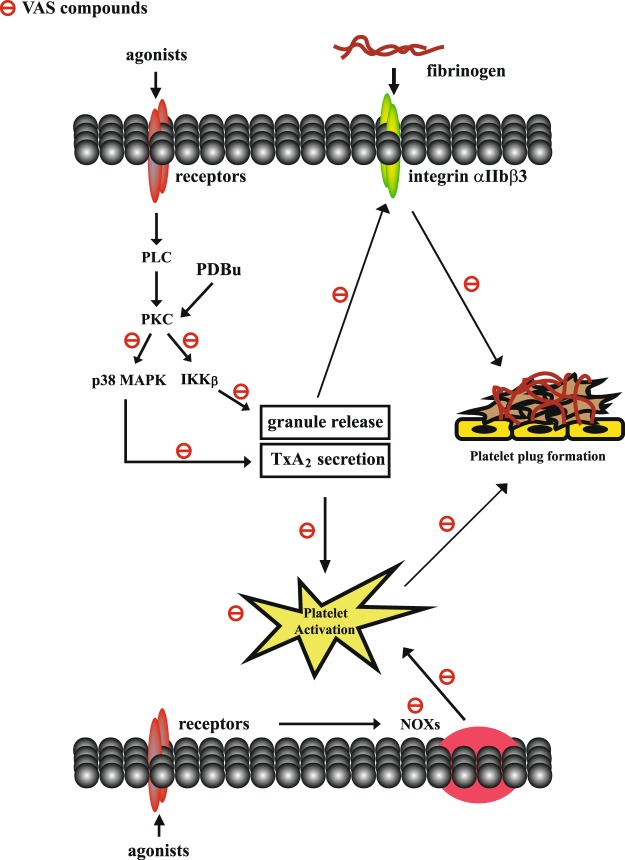
Schematic illustration of VAS compounds-mediated inhibition of platelet activation and thrombus formation.

To the best of our knowledge, in addition to inhibiting NOX activity, the present study demonstrated that VAS2870 and its analog VAS3947 inhibited platelet aggregation, granule release, calcium mobilization, and GPIIbIIIa activation, at least in part, through a NOX-independent pathway downstream of PKC, including IKKβ and p38 MAPK (Fig. 7). In addition, VAS compounds prevented thrombus formation *in vivo*, without affecting normal hemostasis (Fig. 7).

VAS compounds, VAS2870 and VAS3947, are considered as pan-NOX inhibitors and are widely used to investigate the role of NOX in various cell types^17^. VAS2870 specifically inhibits all forms of NOX^17^ and does not interact with ROS in an antioxidant manner or interfere with xanthine oxidase and eNOS^18^. VAS3947, a derivative of VAS2870, shows improved solubility but does not differ in its inhibition profile^5^. Moreover, VAS2870 attenuates platelet-derived growth factor (PDGF)-dependent smooth muscle cell chemotaxis^19^ and reduces oxLDL-induced ROS formation and provasculogenic effects of PDGF-BB on endothelial cells^20,21^ in a NOX-dependent manner. These results indicate that NOX inhibition may be beneficial in cardiovascular diseases. Experimental and clinical evidence has revealed that NOX1 and 2 are involved in platelet activation and thrombus formation^6–11^. Thus, targeting NOX has been suggested to be a potential therapeutic strategy for treating patients with cardiovascular diseases^7^.

However, in the present study, several NOX inhibitors (VAS compounds, ML171, GKT compounds, GSK2795039, DPI and apocynin) were used. We found that, unlike other NOX inhibitors, the VAS compounds VAS2870 and VAS3947 at a concentration of 10 μM markedly abolished platelet aggregation induced by various agonists, including collagen, convulxin, thrombin, and U46619. Moreover, concurrent inhibition of NOX1/2/4 did not inhibit thrombin- or U46619- induced platelet aggregation. These observations suggest that, in addition to serving as NOX inhibitors, VAS compounds may also inhibit platelet aggregation via a NOX-independent manner that may be involved in a convergent point in the platelet activation pathway. Indeed, our data showed that the inhibitory effect of VAS compounds is in association with PKC pathway, a well-known common pathway involved in platelet activation. VAS compounds, but not other NOX inhibitors used in this study, could partially inhibit PDBu-induced platelet aggregation, granule release, calcium mobilization, and GPIIbIIIa activation, but VAS compounds did not reduce PDBu-induced the phosphorylation of PKC substrate, suggesting that VAS compounds act on the PKC downstream signaling pathway. PKC has been reported to regulate platelet granule release, cell spreading, TxA_2_ formation, GP IIbIIIa activation, and finally platelet aggregation^22^. IKKβ has been suggested to conduct PKC-mediated phosphorylation of SNAP-23 that is important for SNARE complex formation and membrane fusion, thereby controlling platelet granule release^13,14^. Moreover, PKC inhibitors could prevent IKKβ activation, but IKK inhibitors did not affect PKC activation^14^, suggesting IKKβ may be the downstream of PKC. In addition, PKC signaling was reported to be required for the secretion of TxA_2_, via MEK/ERK and p38 MAPK pathways^15^. In the present study, we found that PKC-mediated phosphorylation of IKKβ and p38 MAPK was inhibited by VAS compounds as well as Ro 31-8220 in human platelets. These findings suggest that VAS compounds diminish platelet activation, in part, through the inhibition of PKC downstream signaling, including IKKβ and p38 MAPK, which are responsible for granule release and TxA_2_ secretion, respectively. In addition, we did not exclude the possibility that VAS compounds may exert their antiplatelet effects through NOX inhibition. In the present study, we found that PKC activation could induce ROS formation, which could be prevented by the pan-NOX inhibitors VAS compounds (Fig. S6E). Moreover, PKC has been reported to activate NOX in various cells^23^. These observations suggest that VAS compounds may block PKC-mediated ROS formation, in part, through NOX inhibition. However, whether NOX is the downstream of PKC in platelets needs to be further confirmed in the future work. On the other hand, ROS production in activated platelets has been reported to play an important role in regulating platelet activation and formation^24^. However, our data revealed that the inhibition pattern of VAS compounds on platelet aggregation was extremely different from that of other NOX inhibitors. Thus, NOX inhibition may account for one of antiplatelet and antithrombotic effects of VAS compounds.

We also demonstrated that VAS compounds blocked mouse platelet activation induced by collagen and thrombin *in vitro* and significantly prevented thrombosis in the mesenteric microvessels of mice without affecting normal hemostasis. These findings support antiplatelet and antithrombotic effects of VAS compounds, possibly due to their multiple biological activities, including the inhibition of NOXs and PKC downstream pathway.

Although we did not deeply investigate the role of NOXs in platelet activation, several interesting or controversial results were observed in the experiments of platelet aggregation assay of this study. Previously, Delaney *et al*.^9^ reported that NOX1 and NOX2 have differential roles in platelet activation. They reported that NOX1^−/Y^ platelets exhibited selective defects in thrombin- or U46619 (GPCR)-mediated platelet aggregation but no defects in CRP (GPVI)-mediated platelet aggregation^9^. Similar to NOX1^−/Y^ platelets, mouse and human platelets treated with ML171, a NOX1 inhibitor, did not exhibit inhibition of CRP-induced platelet aggregation^11^. Our present data also indicated that ML171 did not affect convulxin (a specific GPVI agonist)-induced human platelet aggregation. However, previous studies and our study revealed that ML171 could inhibit collagen-induced platelet aggregation in mice and humans^6,11^. This discrepancy may be due to the essential role of ROS for platelet activation and aggregation induced by collagen, but not CRP or convulxin^11^. Furthermore, studies have suggested that collagen-induced platelet aggregation is associated with a burst of ROS production^25,26^. These findings indicate that ROS production may be essential in collagen-mediated platelet activation. In addition, NOX2^−/−^ platelets have been found to show a potent inhibition of CRP (0.5 μg/ml)-mediated platelet aggregation but only a partial inhibition of thrombin-mediated platelet aggregation^9^. However, these results appear to be different from that reported by Walsh *et al*.^11^. In their study, NOX2^−/−^ platelets did not exhibit a significant inhibition of CRP (0.25 and 1 μg/ml)-mediated platelet aggregation. Moreover, Walsh *et al*.^11^ reported that NOX2^−/−^ platelets did not exhibit a significant inhibition of collagen-mediated platelet aggregation. Our present study also found that GSK2795039, a NOX2 inhibitor, did not prevent collagen-induced platelet aggregation in mice (data not shown), but it could inhibit such aggregation in humans (Fig. 3C). Thus, the differential role of NOX2 in human and mouse platelets should be further clarified. These findings also suggest that the properties of NOX are different in human and mouse platelets. That needs to be further investigated in the future.

In conclusion, the most important findings of this study revealed that, in addition to inhibiting NOXs, VAS compounds inhibited platelet aggregation-induced by various agonists, partly through the inhibition of a NOX-independent pathway downstream of PKC (Fig. 7). In addition, VAS compounds also markedly inhibited thrombus formation and exerted no significant influence of normal hemostasis in mice. These findings also suggest that, with multiple biological activities, VAS compounds may serve as lead compounds for the development of new effective and safe antiplatelet or antithrombotic drugs for treating patients with cardiovascular diseases. If VAS compounds are to be used to develop new antithrombotic drugs, several factors that have been suggested as critical in drug development^27^, including their solubility, membrane permeation, metabolic stability, and efflux reduction, must be evaluated.

## Materials and Methods

### Materials

VAS2870 was purchased from Enzo Life Sciences (Farmingdale, NY, USA). VAS3947 and GKT136901 were purchased from Merck (Darmstadt, Germany) GSK2795039 was purchased from Med Chem Express (Danvers, MA, USA). DPI, apocynin, and GKT137831 were purchased from Cayman Chemical (Ann Arbor, MI, USA). ML171 and L-012 sodium salt were purchased from Tocris Bioscience (Bristol, UK). Thrombin, collagen, U46619, and convulxin were purchased from Chrono-Log (Havertown, PA, USA). PDBu, luciferase/luciferin, and fluorescein sodium were purchased from Sigma (St. Louis, MO, USA). Fura 2-AM was purchased from Molecular Probe (Eugene, OR, USA). Fluorescein isothiocyanate (FITC)-conjugated anti-P-selectin and PAC-1 antibodies were purchased from Biolegend (San Diego, CA, USA). The anti-phospho-(ser) PKC substrate and anti-phospho-p38 MAPK (Ser^180^/Tyr^182^) polyclonal antibodies (pAbs) and anti-IKKβ and anti-p38 MAPK monoclonal antibodies (mAbs) were purchased from Cell Signaling (Beverly, MA, USA). The anti-pleckstrin (p47) pAb was purchased from GeneTex (Irvine, CA, USA). The anti-phospho- IKKβ (Tyr^188^) pAb was purchased from Abcam (Cambridge, UK). The Hybond-P polyvinylidene difluoride (PVDF) membrane, an enhanced chemiluminescence (ECL), and the horseradish peroxidase (HRP)-conjugated donkey anti-rabbit and sheep anti-mouse immunoglobulin G (IgG) were purchased from GE Healthcare Life Sciences (Buckinghamshire, UK). All drugs (NOX inhibitors) were dissolved in DMSO and stored at 4 °C until use.

### Preparation of washed human platelets

This study was approved by the Taipei Medical University-Joint Institutional Review Board (TMU-JIRB-No. N201701045) and conformed to the principles outlined in the *Declaration of Helsinki*. All volunteers provided informed consent. Human platelet suspensions were prepared as previously described^28^. In brief, blood samples were drawn from healthy volunteers who had taken no medicine, such as aspirin and other NSAIDs or thienopyridines, during the preceding 2 weeks into plastic tubes (polypropylene) and was mixed with an acid-citrate-dextrose (ACD) solution (9:1, v/v). After centrifugation for 10 min, the upper layer containing platelet-rich plasma (PRP) was carefully collected and then supplemented with prostaglandin E_1_ (0.5 μM) and heparin (6.4 IU/ml). After centrifugation, the platelet-poor plasma was discarded, platelet pellets were washed twice, and washed platelets were then suspended in Tyrode’s solution containing 3.5 mg/ml bovine serum albumin (BSA) to obtain platelet suspension. The final concentration of Ca^2+^ in platelet suspension was 1 mM.

### Platelet aggregation

A turbidimetric method was applied to measure platelet aggregation by using a Lumi-Aggregometer (Payton, Scarborough, Ontario, Canada)^28^. In brief, platelet suspensions (3.6 × 10^8^ cells/ml) were pretreated with NOX inhibitors for the indicated concentrations or 0.1% DMSO (an isovolumetric solvent control) for 3 min prior to agonist administration. The platelet aggregation was recorded for 6 min (collagen, convulxin, thrombin, and U46619) or 30 min (PDBu).

### Immunoblotting study

This method was performed as previously described^29^. In brief, platelet suspensions (1.2 × 10^9^ cells/ml) were pretreated with VAS compounds (10 μM), Ro 31-8220 (2 μM) or 0.1% DMSO for 3 min prior to PDBu (150 nM) administration for 20 min to stimulate platelet activation. After the reaction, the platelets were immediately resuspended in 200 μl of a lysis buffer for 1 h. Samples were centrifuged at 5000 × *g* for 5 min. 80 μg of extracted proteins were separated through 12% sodium dodecylsulfate-polyacrylamide gel electrophoresis; The separated proteins were then electrotransferred onto PVDF membrane through semidry transfer (Bio-Rad, Hercules, CA, USA). Membranes were blocked with 5% BSA in TBST (10 mM Tris-base, 100 mM NaCl, and 0.01% Tween 20) for 1 h, and then stained with various specific primary antibodies (diluted 1:1000 in TBST). Membranes were incubated with HRP-conjugated anti-mouse or -rabbit IgG (diluted 1:3000 in TBST) for 1 h. Immunoreactive bands were developed using the ECL kit and quantified using videodensitometry (Bio-Profil; Biolight Windows Application V2000.01, Vilber Lourmat, France).

### ATP release and calcium mobilization measured using a microplate reader

Luciferase/luciferin and Fura 2-AM were used to detect ATP release and calcium mobilization, respectively. This method was described previously^29^. In brief, platelet suspensions (3.6 × 10^8^ cells/ml) were pretreated with luciferase/luciferin or 5 μM Fura 2-AM, and then with VAS compounds (2–10 μM) or 0.1% DMSO for 3 min prior to PDBu administration. The reaction was allowed to proceed for 30 min and the intensity of luminescence was recorded every minute using a Synergy H1 microplate reader (BioTek).

### Flow cytometry

This experiment was performed as described previously^29^. In brief, platelet suspensions (1 × 10^6^ platelets/ml) were pretreated with VAS compounds (2–10 μM) or 0.1% DMSO for 3 min prior to PDBu administration in glass cuvettes at 37 °C. After the reactions for 20 min, platelet suspensions were fixed and labeled with FITC–P-selectin or FITC–PAC-1 antibodies for 30 min to detect P-selectin expression and GPIIbIIIa activation, respectively. After centrifugation and washing, platelet pellets were resuspended with 1 ml of phosphate-buffered saline and then immediately analyzed in a Becton Dickinson flow cytometer (FACScan Syst., San Jose, CA, USA). The platelets were identified and gated by their characteristic forward and side scatter properties and the number of events was stopped at 10,000 counts. All of the experiments were performed at least three times to ensure reliability.

For the competitive binding assay of GP IIbIIIa, platelet suspensions (1 × 10^6^ platelets/ml) were preincubated with VAS compounds (10 μM) or 0.1% DMSO for 3 min prior to FITC–triflavin (2 μg/ml) administration in glass cuvettes at 37 °C. After the reactions for 30 min, a final volume of 1 ml was used for an immediate analysis by a Becton Dickinson flow cytometry.

### Determination of LDH

This assay was performed according to the manufacturer’s protocol from Promega (Madison, WI, USA), as described previously^29^. In brief, LDH release was measured using a CytoTox 96 non-radioactive cytotoxicity assay kit from Promega. Platelet suspensions (3.6 × 10^8^ cells/ml) were pretreated with VAS compounds (10–100 μM) or 0.1% DMSO for 10 min at 37 °C. After centrifugation, the supernatant was collected to measure the LDH level by a Synergy H1 microplate reader (BioTek). The values were recorded at a wavelength of 490 nm. LDH activity was expressed as the percentage of total enzyme activity, which was measured in platelets lysed with 0.5% Triton X-100.

### Detection of ROS formation

Platelet suspensions (3.6 × 10^8^ cells/mL) were incubated with VAS compounds (10 μM) and Ro 31-8220 (2 μM) for 10 min before the addition of L-012 (100 μM), followed by the stimulation with PDBu. Chemiluminescence was detected in a Synergy H1 microplate reader (BioTek, VT, USA).

### Animals

ICR mice (aged 5–6 weeks, weighing 20–25 g, male) were obtained from BioLasco (Taipei, Taiwan). All the procedures involving animals are in accordance with the Guide for the Care and Use of Laboratory Animals (Eighth Edition, 2011) and have been approved by the Affidavit of Approval of Animal Use Protocol-Taipei Medical University (LAC-2017-0193).

### Preparation of washed mouse platelets

Mice were sacrificed with CO_2_ and blood was collected immediately through cardiac puncture into a 1.5 ml tube containing 100 μl of sodium citrate and mixed gently. After centrifugation at 180 × *g* for 5 min, PRP was obtained and mixed with ACD (9:1, v/v). The platelet pellet was obtained after centrifugation at 1300 × *g* for 15 min. Subsequently, the platelet pellet was resuspended with Tyrode’s solution. All the procedures for platelet preparations were conducted at room temperature.

### Fluorescein sodium-induced platelet thrombus formation in mesenteric microvessels of mice

The *in vivo* thrombus formation was measured as described previously^30^. In brief, mice were anesthetized using a mixture containing 75% air and 3% isoflurane maintained in 25% oxygen at a flow rate of 1.5~2 l/min, and the external jugular vein was cannulated with a polyethylene-10 tube for the administration of the dye and drugs. VAS1 (3.7 mg/kg) or VAS2 (4.5 mg/kg) was administered 30 min before sodium fluorescein (15 mg/kg) administration. A segment of the small intestine was placed onto a transparent culture dish and the mesenteric microvessels were observed by a microscopy. Venules (30–40 mm) were selected for irradiation at wavelengths <520 nm to produce a microthrombus by which the time required to occlude the microvessel (occlusion time) was recorded. The dose for mice was accordingly converted from the dose for humans^31^.

### Tail bleeding time

The bleeding time was assessed as described previously^29^. In brief, mice were anesthetized using a mixture containing 75% air and 3% isoflurane maintained in 25% oxygen at a flow rate of 1.5~2 l/min. Saline (control), DMSO (solvent control), VAS1 (3.7 mg/kg), or VAS2 (4.5 mg/kg) was intraperitoneally administrated 30 min before the induction of bleeding by severing the tail 3 mm from the tail tip. The bleeding tail stump was immediately immersed in saline. Subsequently, the bleeding time was continually recorded until no sign of bleeding was observed for at least 10 s. The dose for mice was accordingly converted from the dose for humans^31^.

### Data analysis

Data are expressed as means ± S.E.M. and accompanied by the number of observations (*n*). *n* represents the number of independent experiments conducted with different blood donors. All data were analyzed using analysis of variance (ANOVA) with the Newman–Keuls method as a post-hoc test. *p < *0.05 was considered statistically significant.

## Supplementary information

Supplementary information
