# Supplementary material for: VAS2870 and VAS3947 attenuate platelet activation and thrombus formation via a NOX-independent pathway downstream of PKC
Source: Sci Rep. 2019 Dec 11;9:18852. doi: 10.1038/s41598-019-55189-5 (PMC6906488; doi:10.1038/s41598-019-55189-5)
Supplement: Supplementary file 1 — Supplementary information [file 41598_2019_55189_MOESM1_ESM.pdf]

# VAS2870 and VAS3947 attenuate platelet activation and thrombus formation via a NOX-independent pathway downstream of PKC

**Wan Jung Lu[1,2,3], Jiun Yi Li[4,5], Ray Jade Chen[6,7], Li Ting Huang[1], Tzu Yin Lee[8], and Kuan Hung Lin[2,9,*]**

[1]Department of Medical Research, Taipei Medical University Hospital, 110 Taipei, Taiwan

[2]Department of Pharmacology, School of Medicine, College of Medicine, 110 Taipei Medical University, Taipei, Taiwan

[3]Graduate Institute of Metabolism and Obesity Sciences, College of Nutrition, Taipei Medical University, 110 Taipei, Taiwan

[4]Department of Surgery, Mackay Memorial Hospital, 104 Taipei, Taiwan

[5]Department of Medicine, Mackay Medical College, 252 New Taipei City, Taiwan

[6]Division of General Surgery, Department of Surgery, Taipei Medical University Hospital, 110 Taipei, Taiwan

[7]Department of Surgery, School of Medicine, College of Medicine, Taipei Medical University, 110 Taipei, Taiwan

[8]Graduate Institute of Medical Sciences, College of Medicine, Taipei Medical University, 110 Taipei, Taiwan

[9]Institute of Biomedical Sciences, Mackay Medical College, 252 New Taipei City, Taiwan

Running head: VAS compounds inhibit platelet activation.
Reference numbers: 31
Figure numbers: 7

[*]**Correspondence:** Dr. Kuan-Hung Lin, Institute of Biomedical Sciences, Mackay Medical College, No.46, Sec. 3, Zhongzheng Rd., Sanzhi Dist., New Taipei City 252, Taiwan. Tel: +886-2-26360303 ext. 1726; Fax: +886-2-26360303 ext. 5170, E-mail: linkh@mmc.edu.tw

## Supplementary Figures

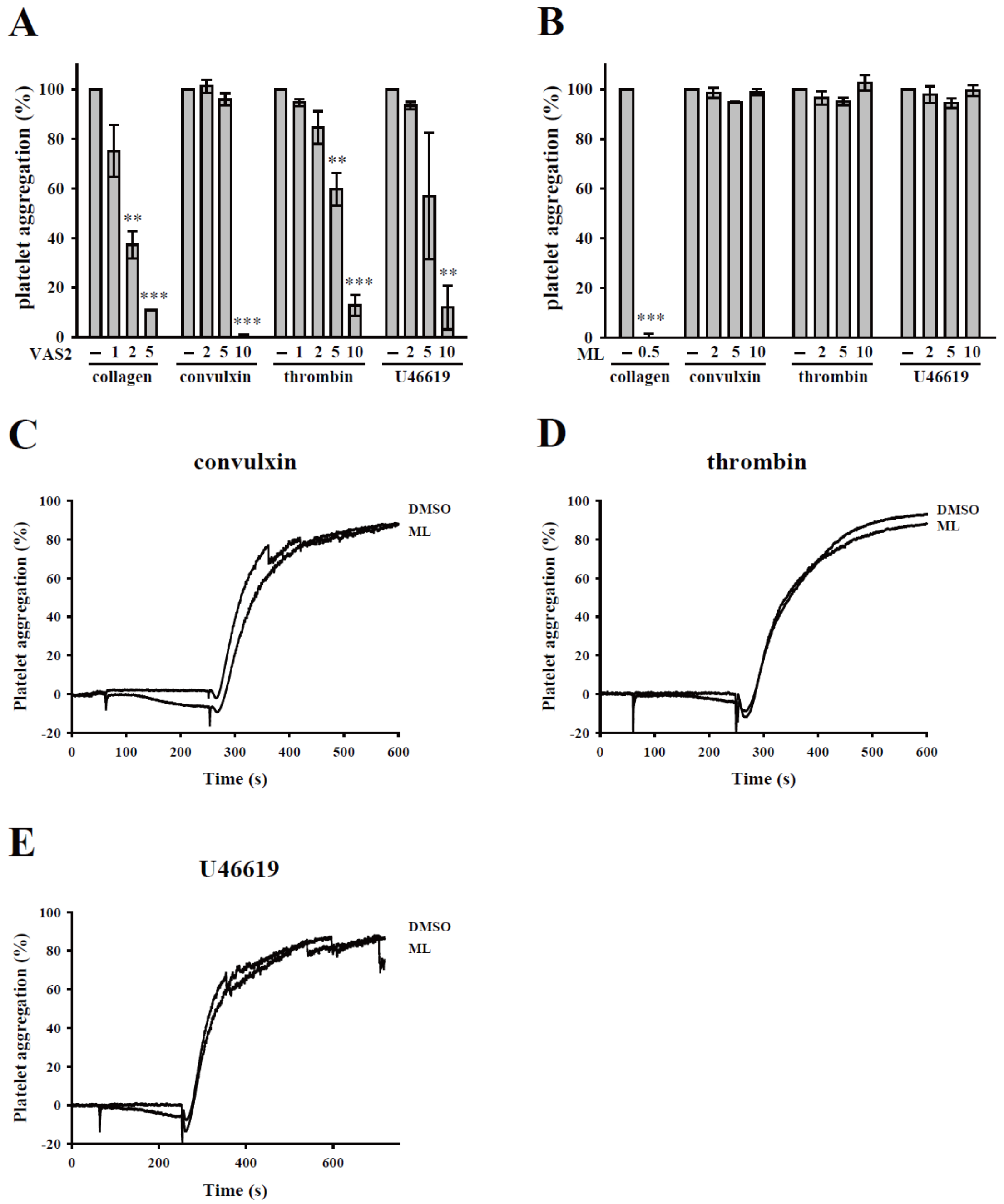


**Supplementary Fig 1.** Effects of VAS2 and ML171 on platelet aggregation in human platelets stimulated by various agonists. Washed platelets (3.6 × $10^8$ cells/ml) were preincubated with DMSO (solvent control), (**A**) VAS2 (1-10 μM), (**B**) ML171 (ML, 0.5-10 μM) or (**C-E**) ML (100 μM) following stimulation with collagen (1 μg/ml), convulxin (10 ng/ml), thrombin (0.02 U/ml), or U46619 (1 μM) to trigger platelet aggregation. Data (**A**, **B**) are presented as means ± S.E.M. ($n$ = 3). $^{**}p < 0.01$ and $^{***}p < 0.001$, compared with the DMSO (solvent control) group. Profiles (**C–E**) are representative examples of three similar experiments.

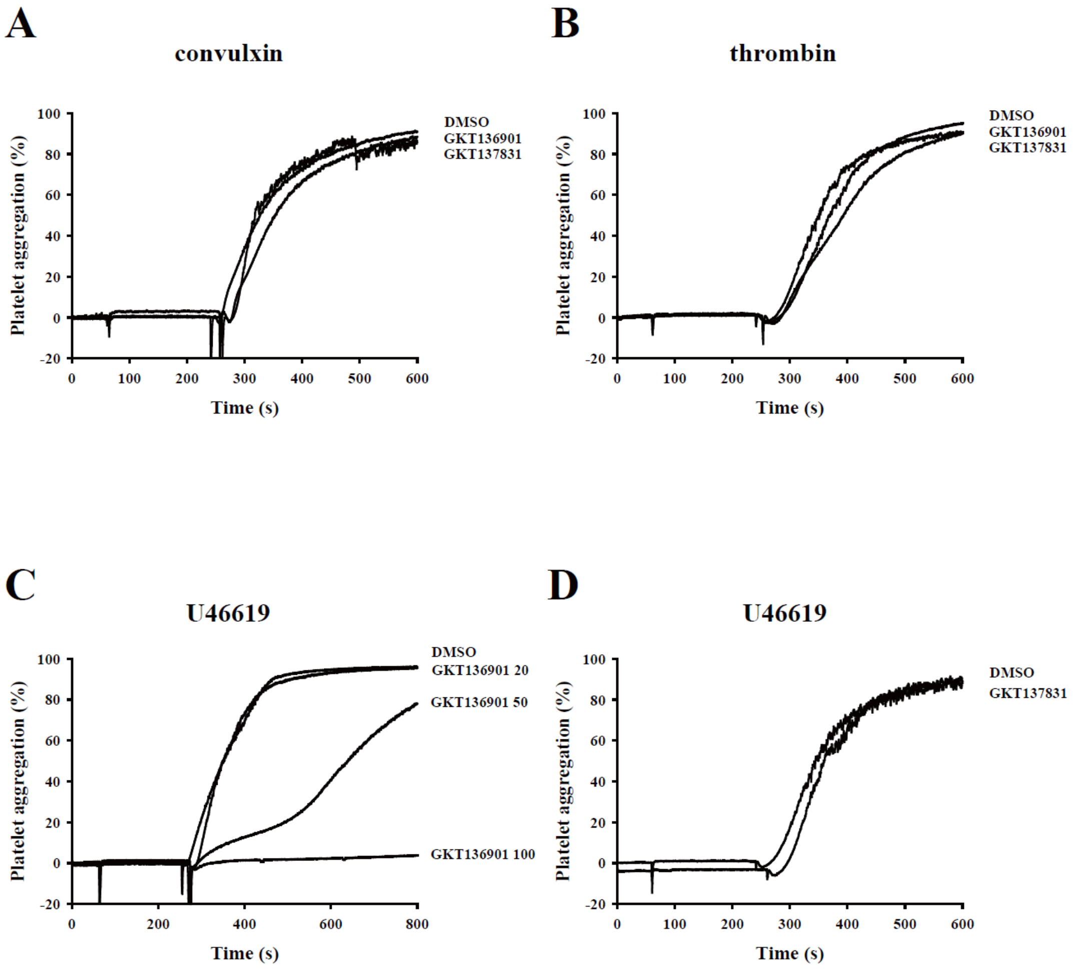


**Supplementary Fig 2.** Effects of GKT compounds (GKT136901 and GKT137831) on platelet aggregation in human platelets stimulated by various agonists. Washed platelets ($3.6 \times 10^8$ cells/ml) were preincubated with DMSO (solvent control), (**A**, **B**, **D**) GKT compounds (100 μM), or (**C**) GKT136901 (20–100 μM) following stimulation with convulxin (10 ng/ml), thrombin (0.02 U/ml) and U46619 (1 μM) to trigger platelet aggregation. Profiles (**A**–**D**) are representative examples of three similar experiments.

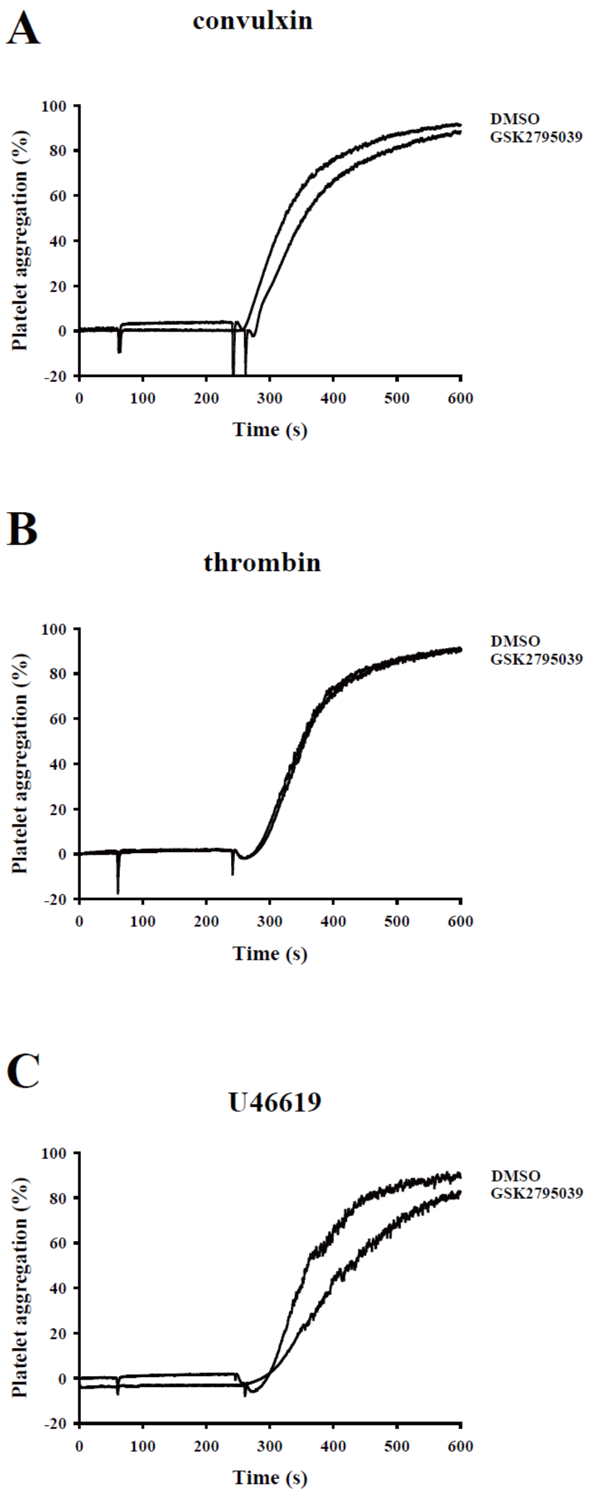


**Supplementary Fig 3.** Effects of GSK2795039 on platelet aggregation in human platelets stimulated by various agonists. Washed platelets ($3.6 \times 10^8$ cells/ml) were preincubated with DMSO (solvent control) or GSK2795039 (100 μM) following stimulation with convulxin (10 ng/ml), thrombin (0.02 U/ml) and U46619 (1 μM) to trigger platelet aggregation. Profiles (**A**–**C**) are representative examples of three similar experiments.

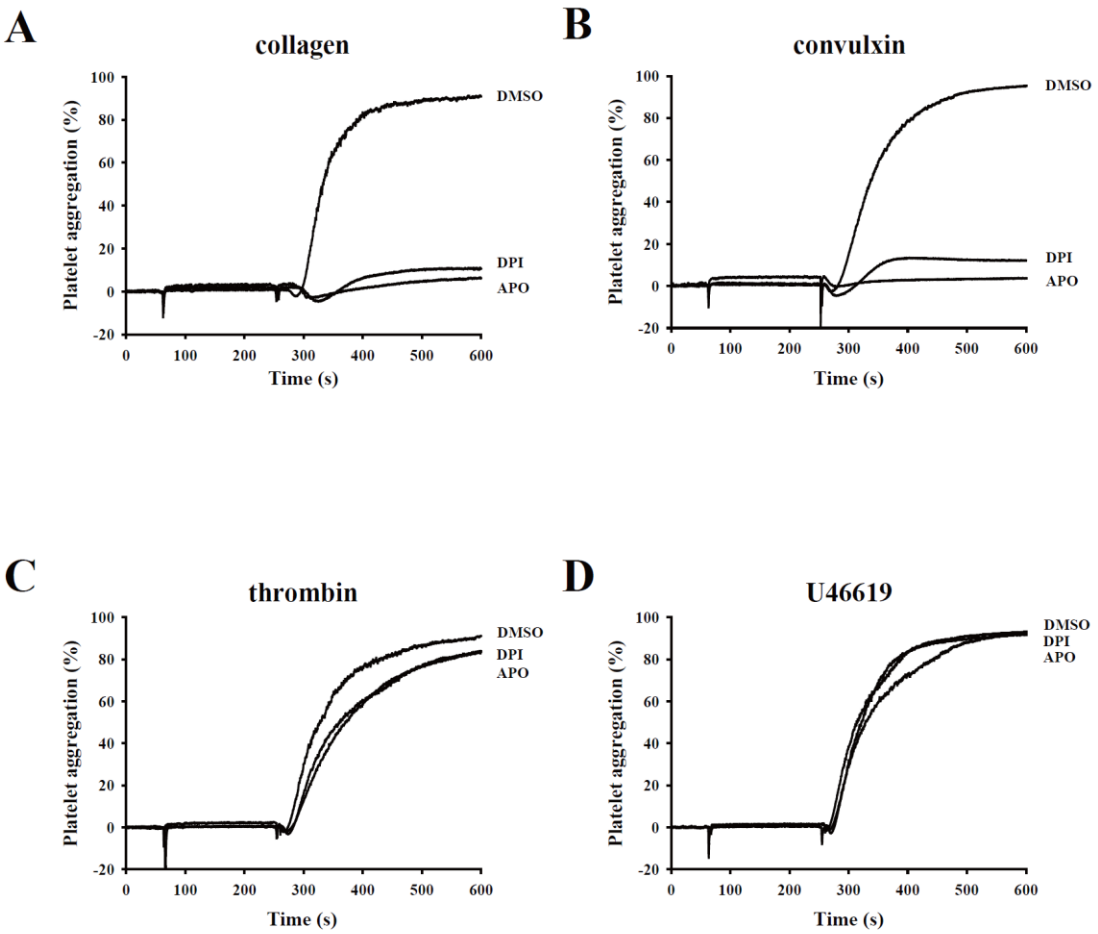


**Supplementary Fig 4.** Effects of DPI and APO on platelet aggregation in human platelets stimulated by various agonists. Washed platelets ($3.6 \times 10^8$ cells/ml) were preincubated with DMSO (solvent control), DPI (100 μM) or APO (500 μM) following stimulation with collagen (1 μg/ml), convulxin (10 ng/ml), thrombin (0.02 U/ml) and U46619 (1 μM) to trigger platelet aggregation. Profiles (**A**–**D**) are representative examples of three similar experiments.

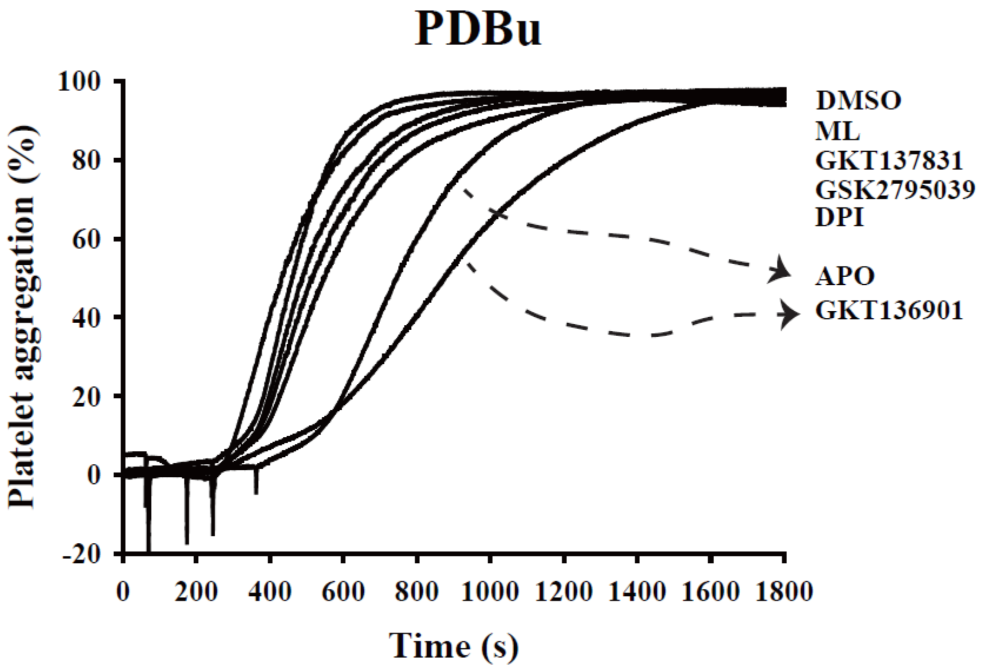


**Supplementary Fig 5.** Effects of NOX inhibitors on platelet aggregation in human platelets stimulated by PDBu. Washed platelets ($3.6 \times 10^8$ cells/ml) were preincubated with DMSO (solvent control), 100 μM ML171 (ML), GKT136901, GKT137831, GSK2795039, and DPI or 500 μM APO following stimulation with PDBu (150 nM) to trigger platelet aggregation. Profiles are representative examples of three similar experiments.

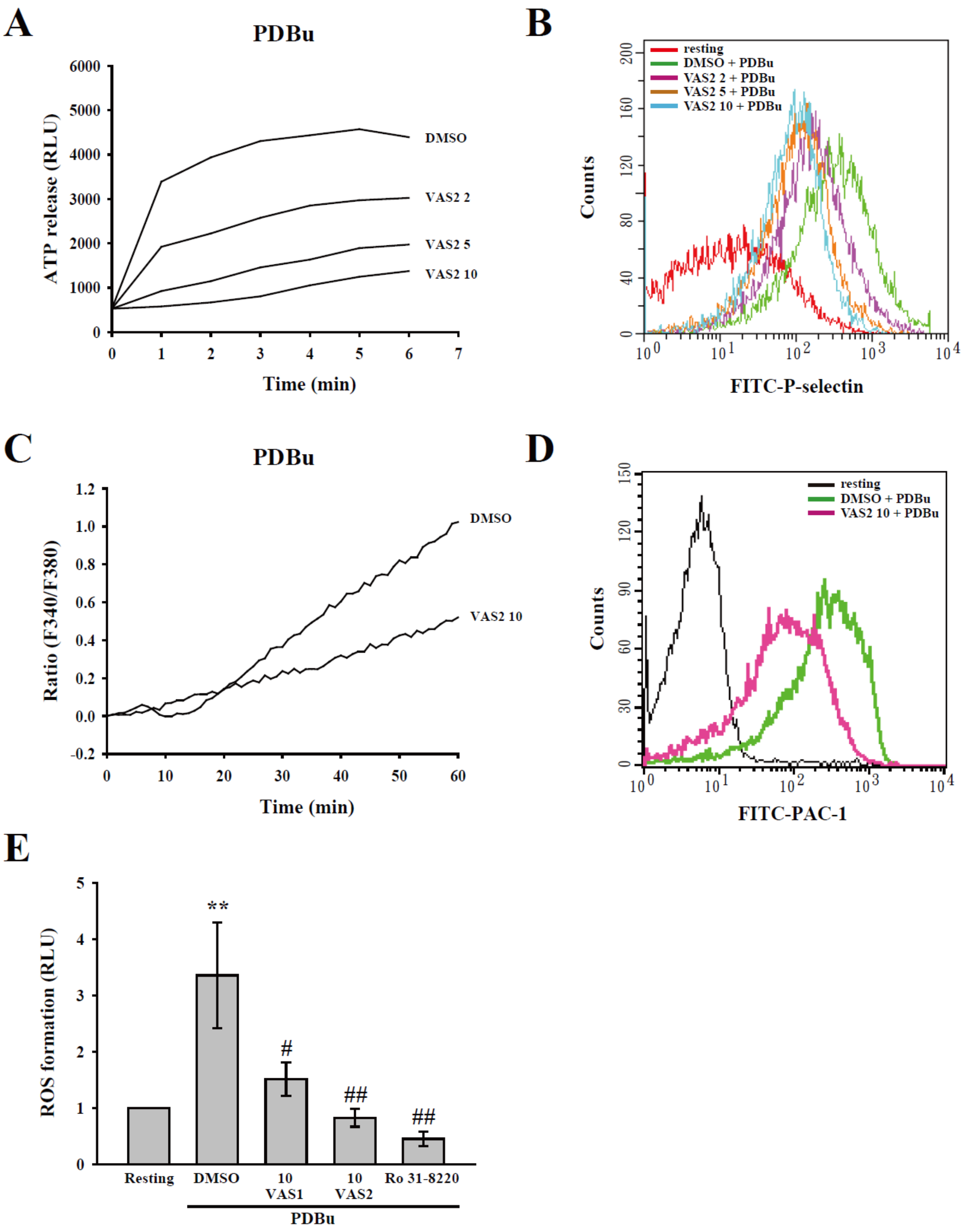


**Supplementary Fig 6.** Effects of VAS1 and VAS2 on granule release, calcium mobilization, GPIIbIIIa activation, and ROS production. Washed platelets were pre-incubated with DMSO (solvent control), VAS1 (10 μM), VAS2 (2, 5, or 10 μM), or Ro 31-8220 (2 μM) before the addition of PDBu to trigger platelet activation. Luciferase/luciferin, FITC-P-selectin antibody, Fura-2, FITC-PAC1 antibody, and L-012 were used to detect (**A**) ATP release, (**B**) P-selectin secretion, (**C**) calcium mobilization, (**D**) GPIIbIIIa activation, and (**E**) ROS production, respectively. Profiles (**A**–**D**) are representative examples of three similar experiments. Data (**E**) are presented as means ± SEM ($n$ = 4). **P < 0.01, compared with the resting group. #P < 0.05 and ##P < 0.01, compared with the PDBu-treated (positive control) group.
